# Tax on Doctors

**Published:** 1879-04

**Authors:** 


### Editorial.

### TAX ON DOCTORS.

"The members of the profession in St. Louis have lately been very much exercised in regard to a special tax levied upon them by the city authorities. An ordinance was passed by the council in 1876, to the effect that each practicing physician should pay a yearly tax of $25.

At a meeting of the profession called in May, 1877, a committee was appointed to collect moneys to defray the expenses of legally resisting such unjust taxation. Dr. Wm. Johnston, as a test case, was sued, and judgment in the first court, rendered against him *proforma* or by agreement; appealed to the second court, judgment rendered in his favor, and the case rested.

About the same time the lawyers went through a similar experience; the Court of Appeals deciding in their favor, the Supreme Court, however, reversed the decision, declaring for the city. Whereupon the collector proceeded to demand payment for license, notifying physicians that $50, for the years 1877–78, were due, failing in the payment of which, arrest and imprisonment seemed the alternative. However, still plucky, the profession, determined by resolution, at two called meetings, held last January, to resist the payment. The dentists were equally interested with the physicians, and conjointly committees were appointed, one to memorialize the State Legislature to remove the power of the City Council to tax doctors, lawyers, dentists and ministers, and the other to address a circular letter to all the physicians throughout the State to instruct their representatives to advocate such an act. As a result of this, the bill has lately passed the lower branch of the Legislature by a large majority, and is now before the Senate with the probabilities of its becoming a law.

While a revenue is necessary to carry on a municipality still this special mode of increasing it were unjust. A merchant's *goods* are taxed because there is *material* representing money; but a physician's capital is his brains, and if this can be legally assessed then why not include the minister, professor, school-teacher, editor, author, architect, civil engineer and all who depend upon their mind, action or brain culture for a subsistence? Where draw the line? Exclusive class legislation were not wise. Besides, if practicing physicians are to be taxed, let it be in amount according to their incomes, not *per capita*.

Many a young and many an impecunious physician, with an income of but a few hundreds, would find a yearly tax of $25 a burden, while more favored ones, with incomes of as many thousands, would not mind it a straw's weight. If assessment is necessary, let it be equalized.

Very properly the government places high duties on luxuries—tobacco, wines, silk, etc., so let it tax the luxury of the knavish, traveling charlatan. While it permits such gross injustice, a blot on the escutcheon of our nation and age, may it look in that direction for income. Quacks and patent medicines of all kinds, if taxed in proportion to the enormity of their evil influences, would go well towards cancelling the municipal obligations, and let the honest, law-abiding physician-citizen go free. If the government will only legislate against and stamp out quackery, no cry will then go up from the doctor if he is called upon to pay an annual stipend of $25, as a tax.

### TAX ON DOCTORS' BUGGIES.

In another matter, also, the city physicians are at opposites with the municipal authorities. A year or more since an ordinance was passed taxing vehicles of all kinds, buggies $3 per year. A few doctors paid, but the large majority have neglected so to do. The legality of the act has been tested and lately decided adverse to the city. Whereat ye poor doctors rejoiceth." S.

The above is clipped from the St. Louis *Courier of Medicine*, and we can well sympathize with our St. Louis brethren, since we have just passed through the same ordeal. The same agency brought relief to each, and, though troublesome to obtain, justice is better late than never. We hope this will be an end of the abortive attempt on the part of municipalities to extort unjust taxes from the professions.

---

THE following will be found serviceable where it becomes necessary to examine urine to perfect the diagnosis of *diabetes mellitus*, and is commendable for its simplicity and accuracy:

| | |
|---|---|
| Cupri sulph. . . . . . . | gr. xxx |
| Potassa (caustic potash. . | gr. xc |
| Aqua distil. . . . . . . | f℥. vj |

M.—To one f℥. of the solution add a *few drops* of the suspected urine, and if sugar be present to any considerable extent, the blue of the former will be changed to an amber color. If sugar exist in only minute quantity, half urine and half solution will be required to make the change of color.

J. H. LOGAN, M.D.,
Prof. Chemistry Atlanta Medical College.

---

DR. A. M. HAMILTON, in his late work on Nervous Diseases, suggests tri-nitro-glycerine as efficacious in aborting epileptic convulsions. A solution of the explosive is made with alcohol as a menstruum, the latter serving also to neutralize the former's explosive qualities. One-tenth of a drop of nitro-glycerine is sufficient to cause the face to flush and to produce other indications of cerebral hyperæmia.

---

THERE are many preparations, such as combinations of beef, iron and strychnia with wine or maltine, that are as convenient as they are efficacious. To physicians in the country, who have not the benefits arising from a contiguous pharmaceutical establishment, these various preparations are decidedly convenient.

Manufacturers are sending out daily samples with a view to establish a trade in this line of goods, and those who try the preparations will find that much time, trouble, and, very often, expense is saved by the investment. Messrs. Reed and Carnrick, Manufacturing Pharmacists, New York, will be found very reliable, and their goods elegantly prepared and of first quality.

